# Ninth Annual Report of the State Board of Health and Vital Statistics of the Commonwealth of Pennsylvania

**Published:** 1895-09

**Authors:** 


Ninth Annual Report of the State Board of Health and Vital Statistics of the Commonwealth of Pennsylvania. Clarence M. Busch, State Printer of Pennsylvania. 1894.

This report represents an exhaustive and attractive summary of the proceedings of the state board of health, as well as the report of the secretary as delegate to the international congress of public health and the American public health association, the circulars and forms pertaining to proclamations in case of epidemics, on the care of the eyes and on dairy hygiene being of special interest.

